# The Role of Prophylactic Cranial Irradiation in Patients With Non-small Cell Lung Cancer: An Updated Systematic Review and Meta-Analysis

**DOI:** 10.3389/fonc.2020.00011

**Published:** 2020-01-23

**Authors:** Lipin Liu, Ting Zhao, Qiuzi Zhong, Jian Cui, Xia Xiu, Gaofeng Li

**Affiliations:** ^1^Department of Radiation Oncology, National Center of Gerontology, Institute of Geriatric Medicine, Beijing Hospital, Chinese Academy of Medical Sciences, Beijing, China; ^2^Department of General Surgery, National Center of Gerontology, Institute of Geriatric Medicine, Beijing Hospital, Chinese Academy of Medical Sciences, Beijing, China

**Keywords:** non-small cell lung cancer, prophylactic cranial irradiation, brain metastasis, survival, toxicity

## Abstract

**Background:** The purpose of this study was to reevaluate the efficacy of prophylactic cranial irradiation (PCI) in non-small cell lung cancer (NSCLC) with the most recent published data and to identify subgroups who may be more likely to gain benefit from PCI.

**Methods:** We searched PubMed, Embase, and Cochrane databases for randomized trials comparing PCI with non-PCI in NSCLC patients. We pooled the data of randomized controlled trials and compared brain metastasis (BM) and overall survival (OS) between PCI group and non-PCI group.

**Results:** Seven studies including 1,462 patients were eligible for the current meta-analysis. Compared to non-PCI group, PCI group achieved decreased BM (RR = 0.37, 95% CI: 0.26–0.52) but similar OS (HR = 1.01, 95% CI: 0.87–1.22). In subgroup analyses of BM, PCI decreased BM for subgroups by pathology (squamous cell carcinoma or non-squamous cell carcinoma) and local treatment modality (surgery or no surgery). However, PCI failed to reduce BM for patients with poor performance status (WHO 2–3). The incidence of PCI related toxicities was low and PCI was well-tolerated by the majority of NSCLC. Low grade neurocognitive function (NCF) decline was reported in NAVLT study and greater deterioration in immediate and delayed recall was reported in RTOG 0214. No significant difference in quality of life (QOL) after PCI was reported.

**Conclusion:** PCI reduces the incidence of BM except for patients with poor performance status. However, PCI fails to prolong OS significantly for NSCLC. An individual patient data meta-analysis may identify patients that could achieve OS prolongation with PCI.

## Introduction

Non-small-cell lung cancer (NSCLC) accounts for ~85% of lung cancer cases (1). NSCLC has a propensity to metastasize to the brain. The incidence of brain metastases (BM) in NSCLC is ~20–40% at some point during their disease course, and the brain is the first site of failure in 15–40% of cases (2, 3). Patients with higher tumor stage, younger age, adenocarcinoma histology have a higher propensity for BM. As a result of increased loco-regional control and prolonged survival with aggressive multimodality treatment, the proportion of patients with BM is increasing. Brain metastases could lead to quality of life (QOL) impairment as well as shortened survival. Although there have been advances in the treatment and management of brain metastases for NSCLC, the prognosis is still poor with a median survival time of <6 months (4).

Prophylactic cranial irradiation (PCI) is the most commonly used therapeutic option to decrease BM so as to prevent the morbidity associated with BM in patients with lung cancer. PCI has been demonstrated to reduce the BM incidence by 50% as well as improve overall survival (OS) for limited-stage small cell lung cancer with complete remission after multi-modality treatment (5). For NSCLC, there have been several randomized controlled trials addressing the value of PCI in the prevention of BM. Previous studies have reported that PCI could reduce the incidence of BM by 50% with uncertain and controversial influence on OS. Recently, a randomized phase III trial RTOG 0214 published its long term updated results suggesting that PCI reduced the 10-years BM (HR = 0.43; 95% CI, 0.24–0.77; *P* = 0.003) while failed to prolong OS significantly (HR = 0.82; 95% CI, 0.63–1.06; *P* = 0.12) for stage III NSCLC. These facts suggest that PCI may not be appropriate for all the patients with NSCLC.

The aim of this study was to re-assess the role of PCI in NSCLC by preforming a systematic review of updated randomized controlled trials and explore the effects of PCI in subgroups with different risk of BM.

## Methods

### Search Strategy

Databases searched included the PubMed, Embase and Cochrane Central Register of Controlled Trials (CENTRAL). The literature search was conducted initially from the date of their inception until May 31, 2019. Search terms included “non-small cell lung carcinoma,” “cranial irradiation,” “survival,” and “randomized controlled trials” (see Supplementary Material). The search was limited to clinical studies. Eligible published trials were also identified from reference lists from RCTs and systematic reviews.

### Selection Criteria

Articles that met the following criteria were included: (1) Study type: RCT; (2) Participants: patients with cytologically or histologically confirmed NSCLC; (3) Intervention: PCI; (4) Reported outcome: BM and OS; (5) Language: English only. Reviews without original data, meta-analyses, animal studies, and studies with abstracts only or duplicated data were excluded.

### Data Extraction and Quality Assessment

Two independent researchers reviewed titles, abstracts, and full text papers and collected data from included full text papers. The following information was extracted from the included RCTs: general information of the included studies, study design, and treatment outcome. If the data of same patient cohort were published in multiple reports, the most recently published data were collected. Discrepancies were resolved by a third researcher.

The quality of RCTs was assessed according to the Cochrane collaboration's tool for the assessment of risk of bias (6). To provide a qualification of risk of bias, the tool consists of seven items including details of the randomization method, allocation concealment, blinding of participants and personnel, blinding of outcome assessment, incomplete outcome data, selective reporting and other sources of bias. Each of the items is scored as “low risk,” “unclear risk,” or “high risk.”

### Statistical Analysis

Statistical analysis was conducted by Review Manager (RevMan) version 5.3 and STATA 12.0. Relative risks (RR) and 95% confidence intervals (CI) for BM were calculated based on the number of BM events as well as group totals. Hazard ratios (HR) and 95% CI for OS were extracted from the research article or derived by the use of other available data according to the method proposed by Tierney et al. (7). For the study by Umsawasdi (8) presenting Kaplan-Meier survival curves, Engauge Digitizer was used to derive HR for OS from the Kaplan-Meier graph (7).

Subsequently, random-effect model was used to calculate the pooled RR or HR and 95% CI for each treatment outcome. Subgroup analysis of BM and OS was conducted to determine whether the results would change according to pathology (squamous cell carcinoma vs. non-squamous cell carcinoma), local treatment modality (prior surgery vs. no prior surgery) and performance status (WHO 0–1 vs. WHO 2–3). Heterogeneity of the studies was assessed by the use of *I*^2^-tests (9). Egger's linear regression test and Begg's rank correlation test were performed to assess publication bias (10, 11). If Egger's test indicates significant publication bias, a sensitivity analysis will be conducted excluding small studies (weight <10%). All statistical tests were two-sided and statistical significance was defined as *P* < 0.05.

## Results

### Literature Search and Methodological Quality Assessment

**Figure 1:**
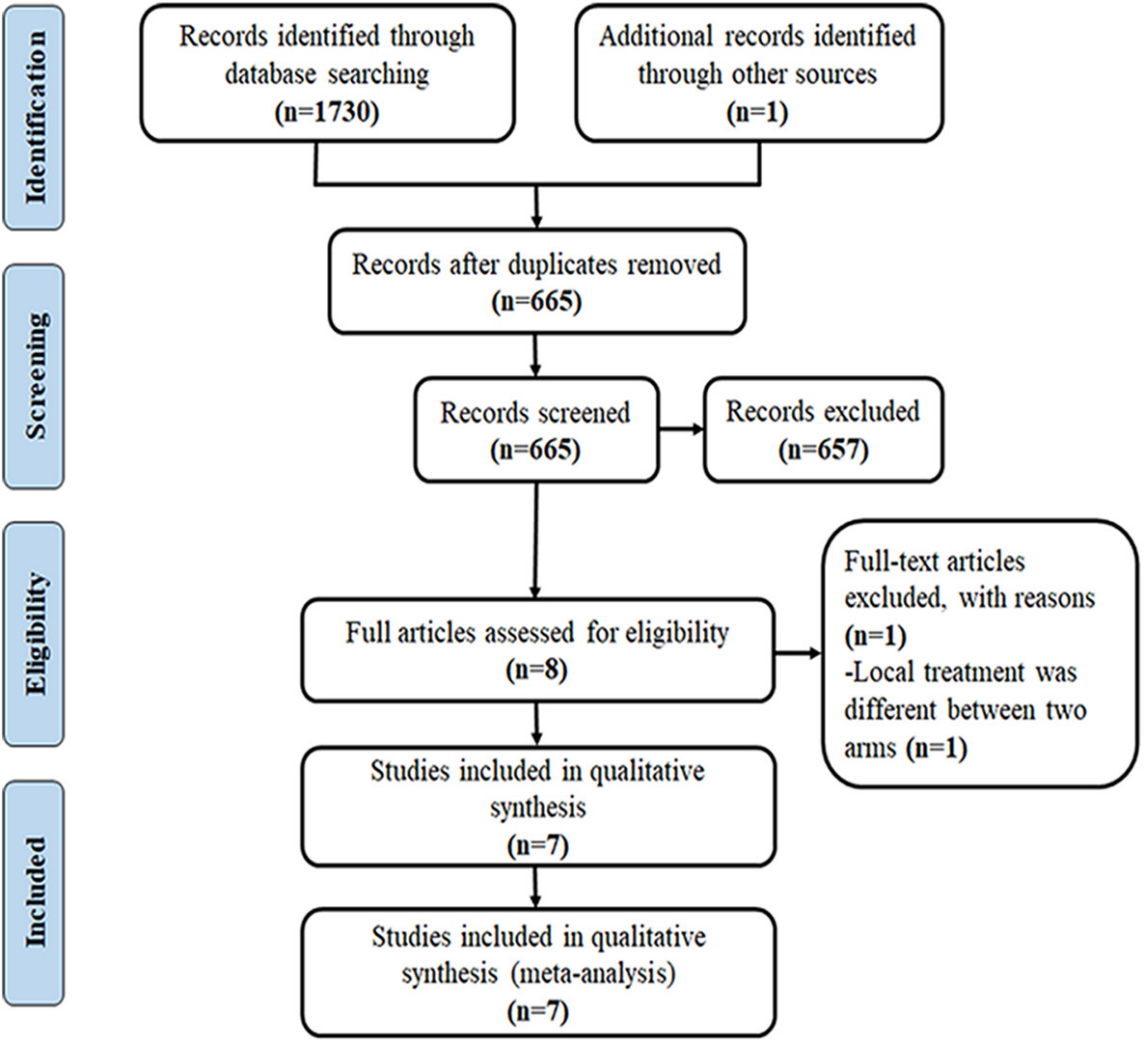
Flow diagram for identification of eligible studies.

Citation selection was illustrated in Figure 1. A total of 1,371 publications were yielded after electronic literature search. After removing the duplicated and irrelevant records, eight full-text articles were assessed for eligibility. After reading the full text, one randomized trial (12) was excluded due to heterogeneous local treatment between the two arms (curative surgery followed by post-operative thoracic radiotherapy vs. chemotherapy and concurrent chemoradiotherapy followed by thoracic surgery).

**Figure 2:**
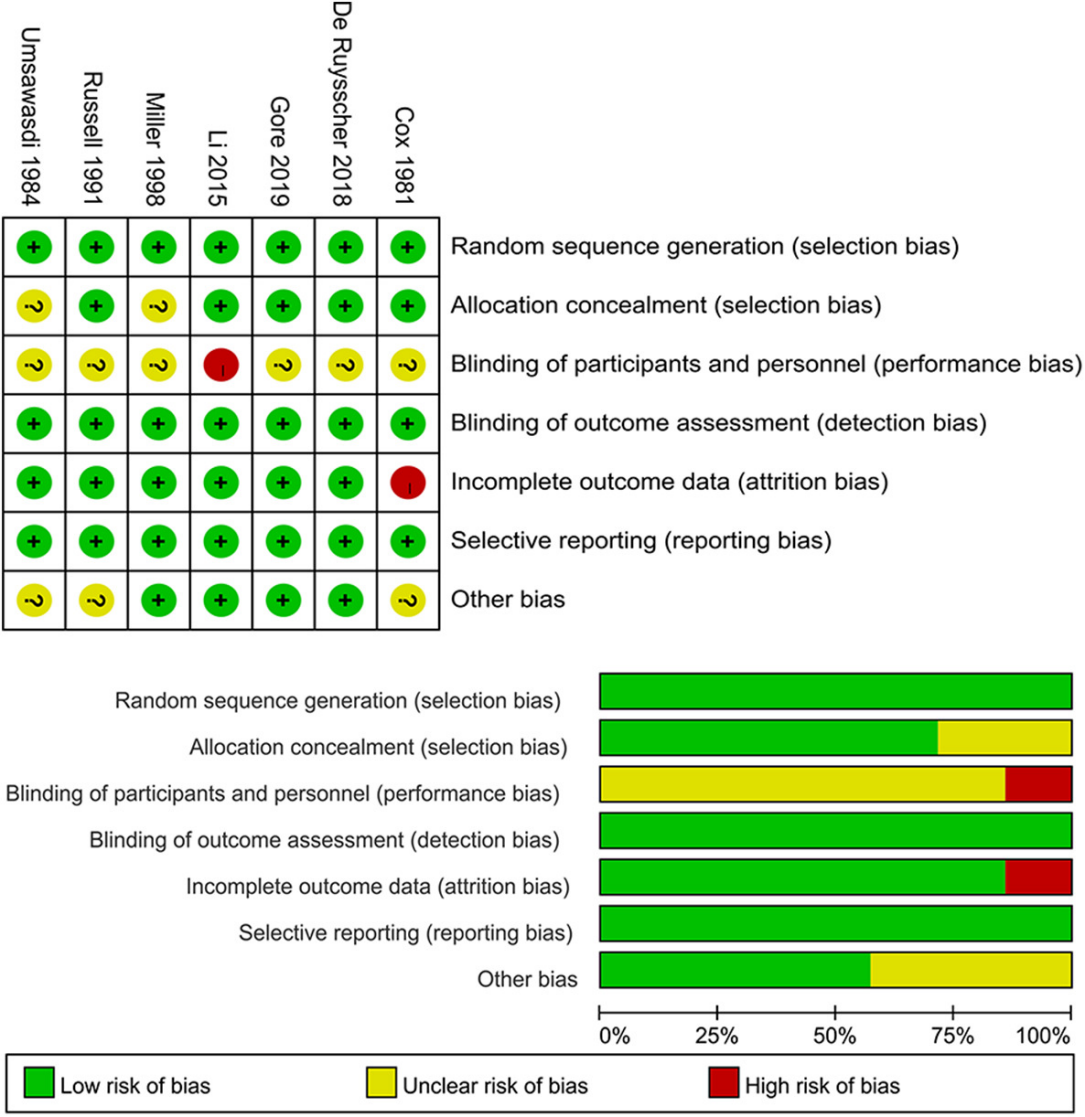
Methodological quality assessment of the included randomized controlled trials (upper: risk of bias graph; low: risk of bias summary).

The results of methodological quality assessment are presented in Figure 2. In terms of randomization sequence generation, all included studies were judged to be at low risk. Furthermore, allocation concealment was adequate in five of the included studies (13–17). The reviewers judged the study of Li et al. (15) to be at high risk in regard to blinding of participants and personnel because the study was an open-label trial. Since all included studies assessed outcomes by objective method, they were judged to be at low risk of introducing detection bias. Due to lack of intention to treat analysis in the study of Cox et al. (13), the study were judged to be at high risk of introducing attrition bias.

### Study and Patient Characteristics

The study and characteristics of the included seven trials are shown in Table 1. The seven included studies reported on a total of 1,462 patients between 1981 and 2019, with 717 in PCI group and 745 in non-PCI group. Several important individual differences exist among the included studies. Firstly, four studies (14, 15, 17–19) enrolled stage III NSCLC patients only, two studies (8, 16) included stage I-III patients, and one study (13) included inoperable NSCLC patients with unclarified stage. Secondly, the local curative treatment preceding PCI differed from one study to another. The patients in study of Cox et al. (13) received radiotherapy alone. Three trials (8, 14, 17) treated patients with curative local treatment consisted with radiotherapy, chemotherapy and surgery. One trial treated patients with surgery and adjuvant chemotherapy (15). Patients enrolled in RTOG 8403 (16) were administered with chest radiotherapy alone or surgery followed by chest radiotherapy. And one trial (18, 19) treated patients with combination of chemo-radiotherapy and radiotherapy alone. Thirdly, brain imaging method differed between trials. Most studies mandated a brain scan by radionuclide (8, 13), CT (8, 14, 16), or MRI (14, 15, 17) prior to study entry. However, one study (18, 19) did not give any information regarding pretreatment brain imaging. Fourthly, the most commonly used prescription dose for PCI was 30 Gy (in 10 or 15 fractions) whereas some patients received 37.5 Gy in 15 fractions, 20 Gy in 10 fractions, or 36 Gy in 18 fractions.

**Table 1:** Characteristics of included studies.

**Study**	**Year**	***N***	**Stage**	**Primary treatment**	**Brain imaging**	**PCI dose**	**Brain metastases (%)**			**Median survival (months)/overall survival % at [X year(s)]**		
							**PCI**	**Control**	***P***	**PCI**	**Control**	***P***
VALG	1981	281	Inoperable	RT alone	Radionuclide scan	20 (2 Gy^*^10)	7/136 (5.1%)	16/145 (11%)	0.038	8.2 m	9.7 m	0.5
MDACC	1984	97	I–III	Tri-modality (Surgery + RT + CT)	Radionuclide scan/CT scan	30 (3 Gy^*^10)	2/46 (4.3%)	14/51 (27.5%)	0.002	22% (3 y)	23.5% (3 y)	NA
RTOG 8403	1991	187	I–III	RT alone Surgery and RT	CT	30 (3 Gy^*^10)	8/93 (8.6%)	18/94 (19.1%)	0.1	8.4 m 40% (1 y) 13% (2 y)	8.1 m 44% (1 y) 21% (2 y)	0.36
SWOG	1998	226	III	Chemo-RT RT alone	Unclear	30 (2 Gy^*^15) 37.5 (2.5 Gy^*^15)	1/111 (0.9%)	13/115 (11.3%)	0.003	8 m	11 m	0.004
RTOG 0214	2019	340	III	Tri-modality (Surgery + RT + CT)	MRI scan	30 (2 Gy^*^15)	20/163 (12.3%)	40/177 (22.6%)	0.003	28.8 m 24.7% (5 y) 17.6% (10 y)	25.2 m 26.0% (5 y) 13.3% (10 y)	0.12
Li	2015	156	IIIA-N2	Surgery-chemo	MRI	30 (3 Gy^*^10)	10/81 (12.3%)	29/75 (38.7%)	<0.001	31.2 m 44.5% (3 y) 27.4% (5 y)	27.4 m 38.7% (3 y) 22.8% (5 y)	0.310
NVALT-11	2018	175	III	Tri-modality (Surgery + RT + CT)	MRI/CT	36 (2 Gy^*^18) 30 (2.5 Gy^*^12) 30 (3 Gy^*^10)	6/87 (7.0%)	24/88 (27.2%)	<0.001	24.2 m	21.9 m	0.56

* *All inoperable patients; stage nor clear*.

### Outcomes

#### Brain Metastasis

**Figure 3:**
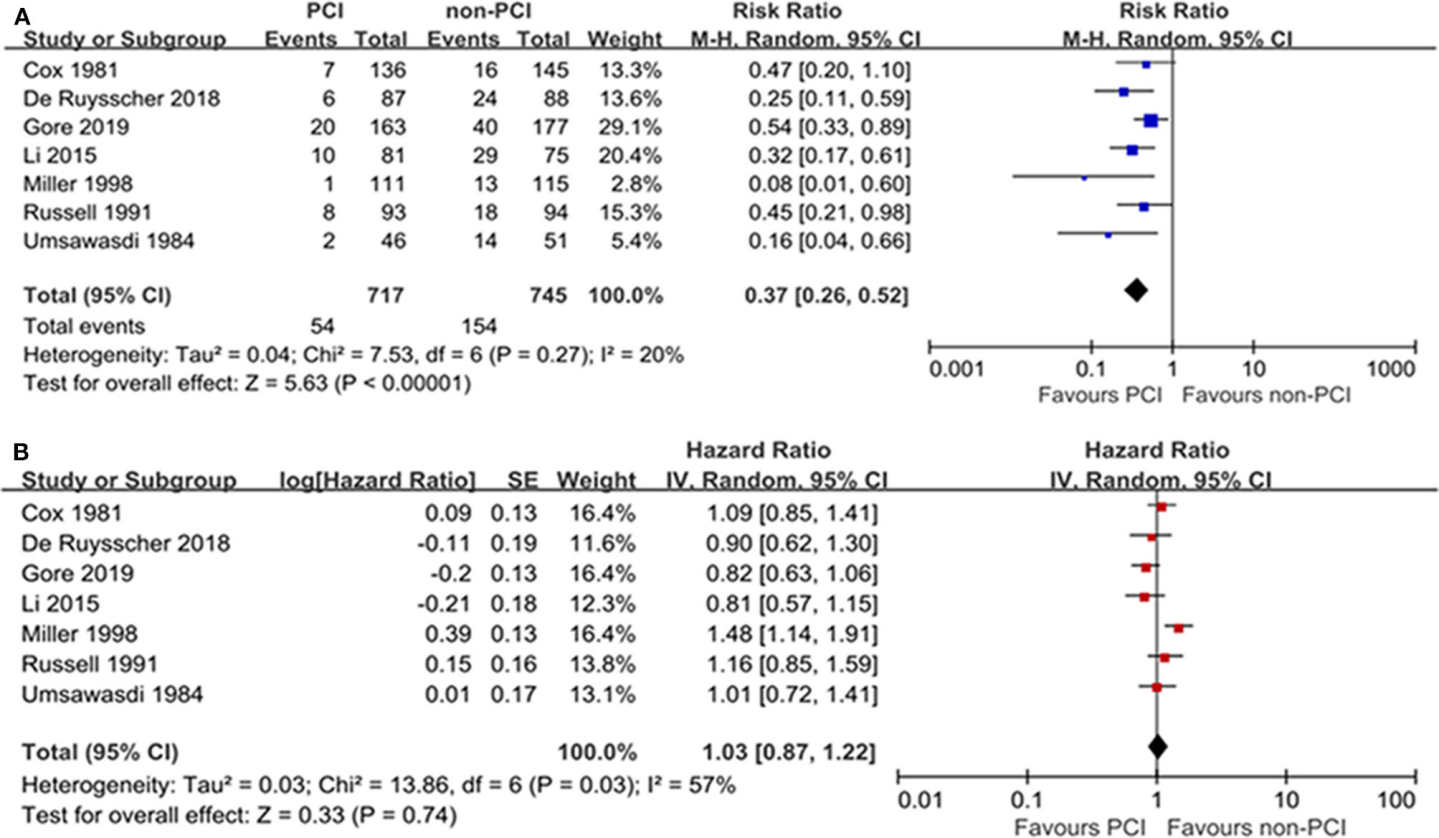
Forest plots of relative ratio (RR) on brain metastasis **(A)** and hazard ratio (HR) on overall survival **(B)**.

As to the effect of PCI on BM, the majority of included studies demonstrated a significant decrease in the incidence of brain metastases brought by PCI. Taken all included trials together, PCI compared with non-PCI was correlated with a significant reduction on BM (RR: 0.37; 95% CI: 0.26–0.52) based on random-effect model according to the results of the heterogeneity test (*I*^2^ = 20%, *P* = 0.27). The forest plot for BM was shown in Figure 3A. Egger's test suggested significant publication bias (*P* = 0.018) while Begg's test indicated no significant publication bias (*P* = 0.072). Sensitivity analysis excluding small studies (8, 18, 19) (weight < 10%) showed that PCI could decrease the risk of BM (HR = 0.42; 95% CI: 0.31–0.57) without significant publication bias assessed by Egger's test (*P* = 0.311).

#### Overall Survival

The OS outcome was measured in terms of the HR of the PCI group compared with the non-PCI group. The combined result revealed no significant OS prolongation brought by PCI with a pooled HR of 1.01 (95% CI: 0.87–1.22) (Figure 3B). The heterogeneity between the PCI group and non-PCI group was statistically significant (*I*^2^ = 57%, *P* = 0.03). Sensitivity analysis showed that the heterogeneity was largely due to the study by Millar et al. (18) which revealed an opposite direction of effect (HR = 1.48) from the other 6 trials (HRs from 0.81 to 1.16). As a result, we combined the results by a random-effect model. No significant publication bias was suggested by Egger's and Begg's tests (*P* = 0.635 and 0.734, respectively).

#### Subgroup Analyses

**Figure 4:**
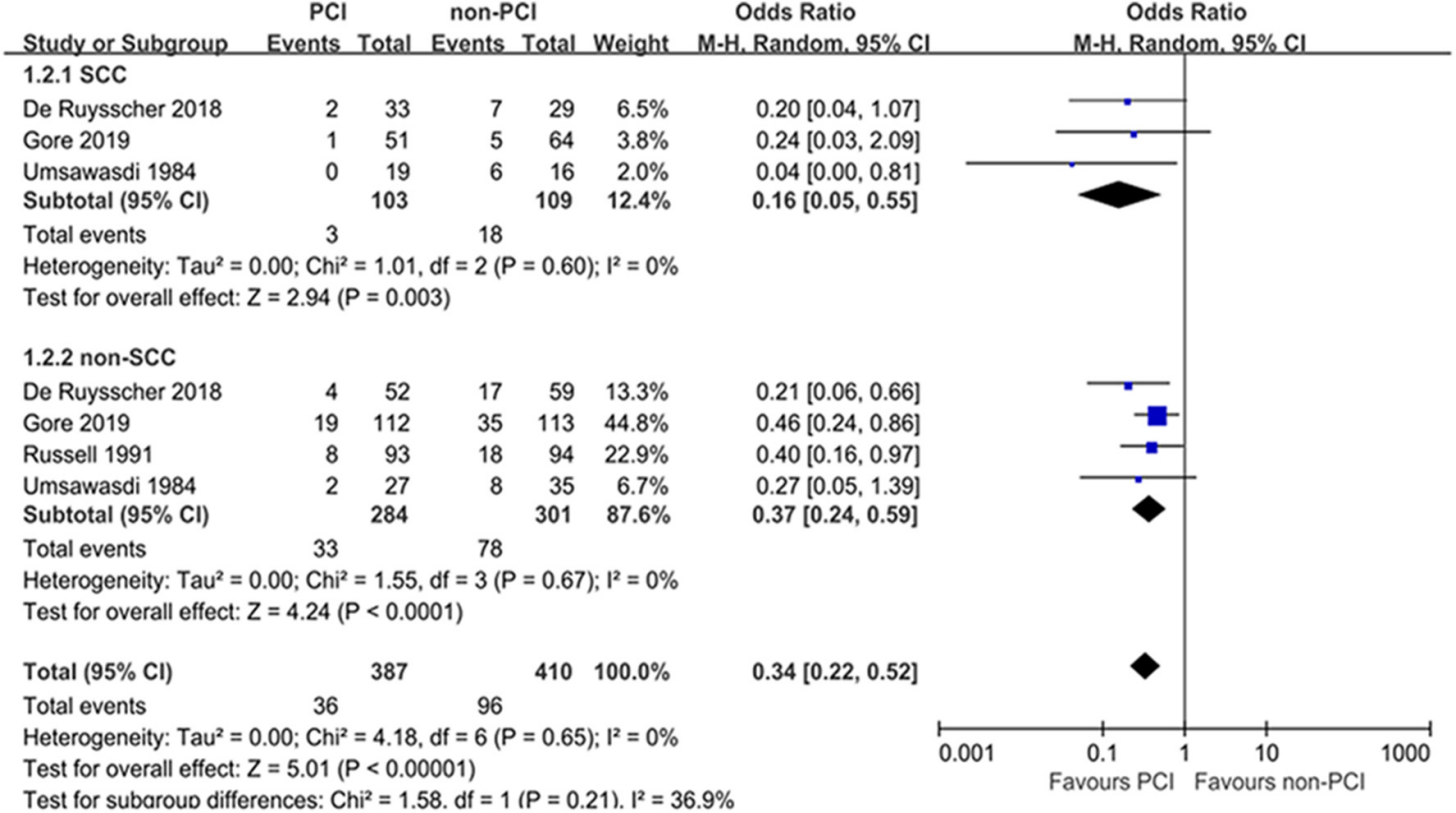
Subgroup analyses for BM according to pathology.

**Figure 5:**
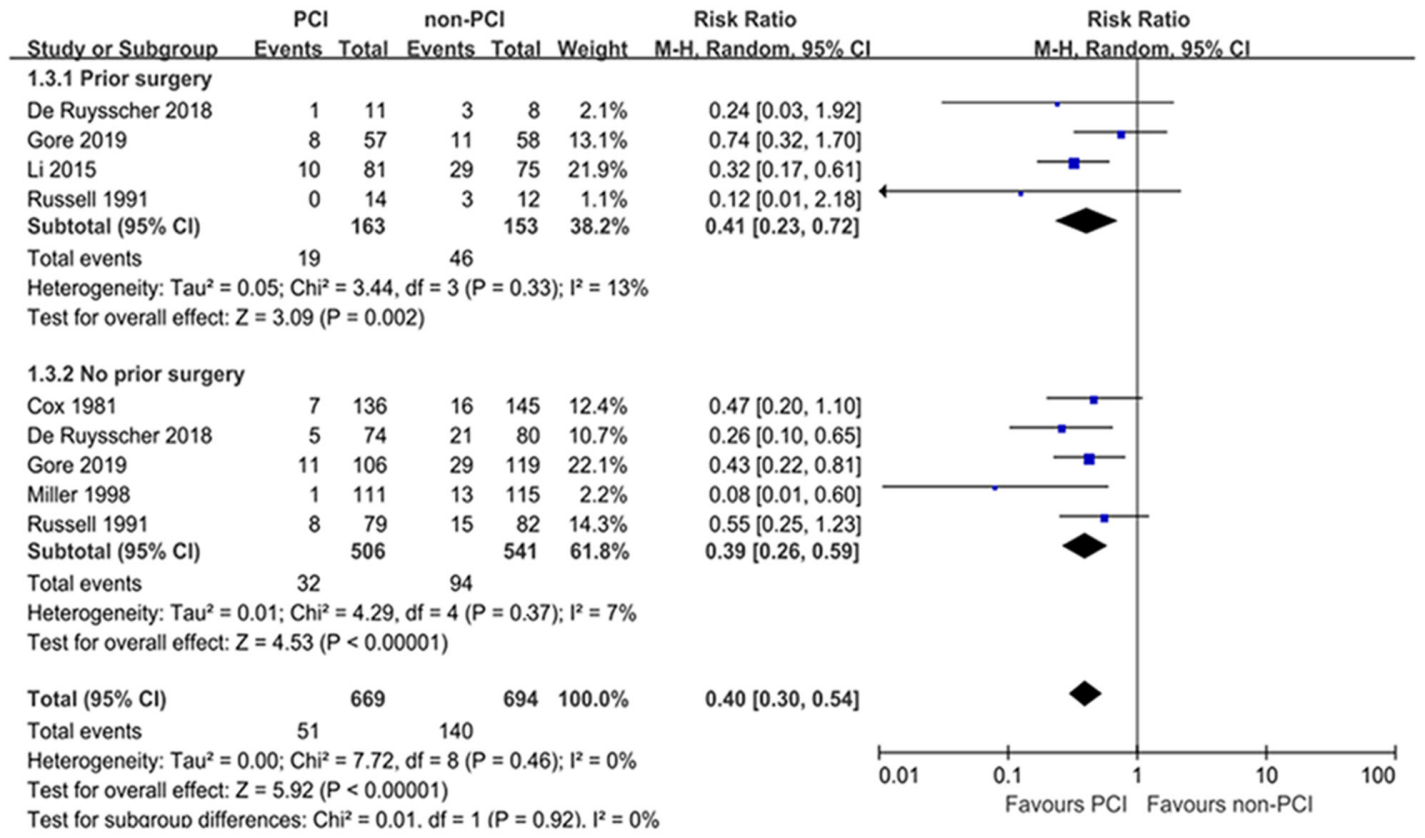
Subgroup analyses for BM according to prior treatment.

**Figure 6:**
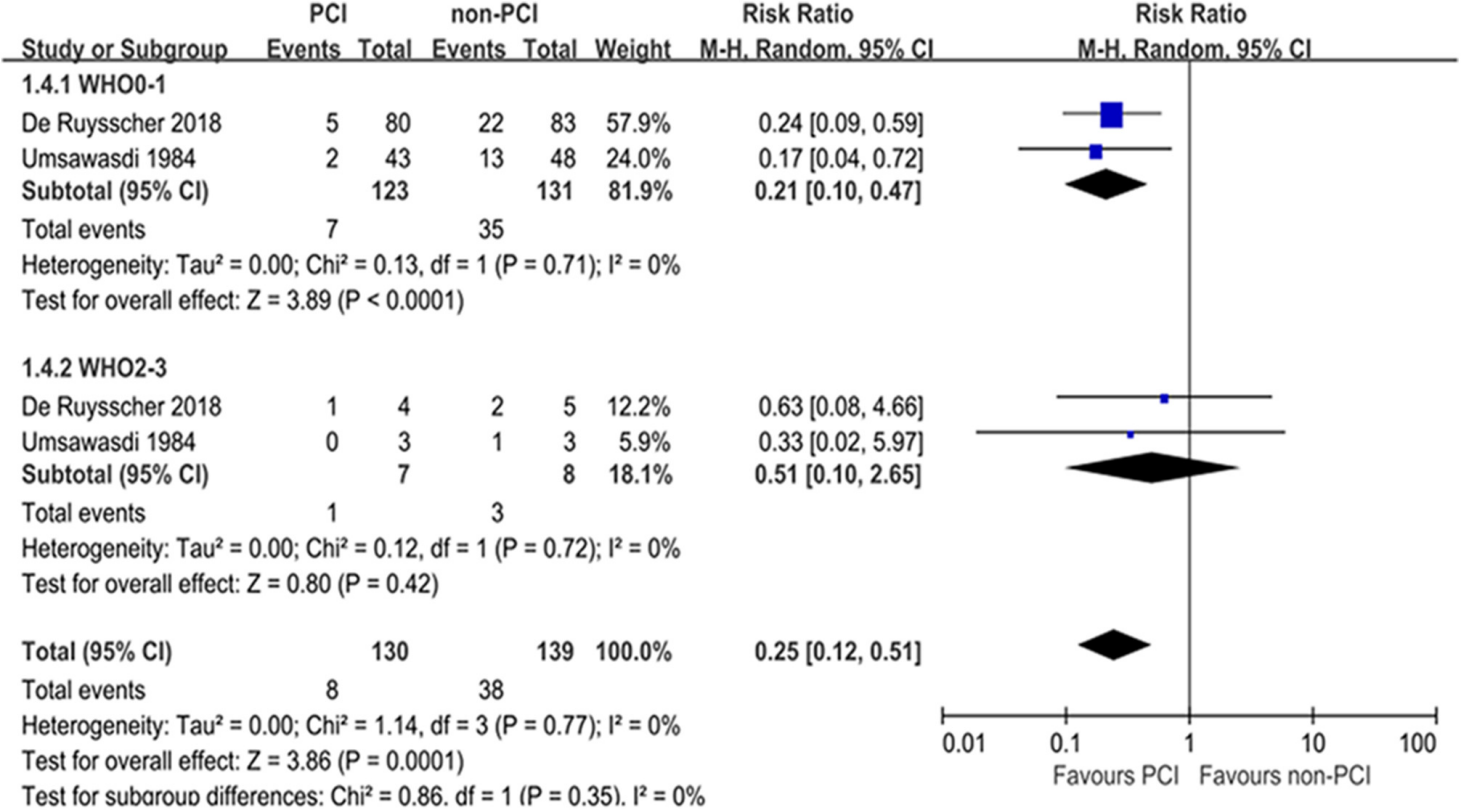
Subgroup analyses for BM according to performance status.

Subgroup analyses for BM were performed according to pathology, prior treatment, and performance status (Table 2). Subgroup analyses demonstrated that the treatment effects of PCI were similar between the predefined subgroups by pathology (Figure 4) and prior treatment to that observed in the primary analysis of BM in the overall population (Figure 5). As shown in Figure 6, PCI could decrease BM by 79% (HR 0.21; 95% CI, 0.10–0.47) for patients with good performance status (WHO 0–1). However, PCI failed to provide a significant beneficial effect on BM (HR 0.51; 95% CI, 0.10–2.65) for patients with WHO 2–3.

**Table 2:** Subgroup analyses of brain metastasis.

**Subgroup**	**No. of studies**	**Pooled RR**	**95%CI**	**Heterogeneity *I*^**2**^; *P*-value**	**P-value for heterogeneity between subgroups**
Pathology					0.21
SCC	3	0.16	0.05–0.55	0%; 0.60	
Non-SCC	4	0.34	0.22–0.52	0%; 0.67	
Prior treatment					0.92
Surgery	4	0.41	0.23–0.72	13%; 0.33	
Non-surgery	5	0.39	0.26–0.59	7%; 0.37	
Performance status					0.35
WHO 0–1	2	0.21	0.10–0.47	0% 0.71	
WHO 2–3	2	0.51	0.10–2.65	0%; 0.72	

#### PCI Related Adverse Events and Quality of Life (QOL)

The summary of PCI related toxicities and QOL are shown in Table 3. Most included studies except for that of Cox et al. (13) reported data on the PCI related toxicities while only few studies assessed QOL (14, 15, 20). The PCI related acute toxicities were as follows: alopecia (grade 1–2 in 42%) (8, 14), headache (grade 1–3 in 27–38% and grade 3 in 1%) (14, 15), fatigue (grade 1–3 in 22–64% and grade 3 in 2–15%) (14, 15, 17), skin toxicity (grade 1–2 in 5%) (8, 15), and nausea and vomiting (grade 1–3 in 23–35%, and grade 3 in 5%) (14, 15, 17).

**Table 3:** PCI related adverse events and QOL results of included studies.

**Study**	**PCI related toxicities**	**Neuropsychological tests and results**	**QOL instruments and results**
MDACC	Acute toxicitiy: one patient develped transient memory loss for 2.5 weeks Late toxicity: none	Not reported	Not reported
RTOG 8403	Acute toxicity: epilation and skin reactions Late toxicity: none	Not reported	Not reported
SWOG	Not reported	No excessive neurological toxicity with PCI, but the definition of neurological toxicity was not stated	Not reported
RTOG 0214	Acute toxicity: constitutional (grade 1–2), gastrointestinal (grade 1), dermatologic (grade 2), fatigue (grade 3), ataxia (grade 3), dyspnea (grade 3), depression (grade 3–4), hematologic (grade 3), pain (grade 3) Late toxicity: grade 3 dyspnea, syncope, weakness, fatigue and soft tissue necrosis	MMSE, HVLT, and ADLS No significant differences in global cognitive function (MMSE), but there was a significant decline in memory (HVLT) at 1 year	EORTC QLQ-C30 + BN20 No significant differences in QOL after PCI
Li	Acute toxicity: headache (grade 1–2, 26%; grade 3, 1%), nausea or vomiting (grade 1–2, 23%), fatigue (grade 1–2, 13%; grade 3, 2%), skin toxicity (grade 1–2, 5%), insomnia (grade 2, 2%) Late toxicity: mild headache/slight lethargy (22.2%), moderate headache/great lethargy (11.1%), severe heacache (2.5%); grade 3 skin strophy (1%), grade 3 fatigue (1%)	Not reported	FACT-L questionaire No significant differences were noted in deterioration rate for QOL and symptoms between the two groups
NVALT-11	Alopecia, gatigue, headache	CTCAE 3.0 Memory impairment (grade 1–2) and cognitive disturbance (grade 1–2)	EORTC QLQ-C30 + BN20 and EuroQoL 5D At 3 months after PCI, QOL was worse in the PCI arm. At 6–18 months, QOL was simialr between both arms. At 24–48 months, there was a slight and non-significant advantage in QOL in the observation arm

In the study of Umsawasdi et al. (8) and Russell et al. (16), no late complication were noted. However, in the updated report of RTOG 0214, five patients (3%) experienced grade 3 late toxicity, including dyspnea, syncope, weakness and fatigue, and soft tissue necrosis (17). Li et al. reported late toxicities as follows: mild headache or slight lethargy (22.2%), moderate headache or great lethargy (11.1%), severe headaches (2.5%), grade 3 skin atrophy (1%), and fatigue (1%) (15).

As for neurocognitive adverse events, two included studies addressed this issue. The trial NAVLT reported high incidence of grade 1–2 memory impairment (30 vs. 8%) and cognitive disturbance (18.5 vs. 3.5%) in the PCI group compared with observation group (14). However, RTOG 0214 reported no neurocognitive function deterioration by the tool of Mini-Mental Status Examination (MMSE) while greater deterioration in immediate recall and delayed recall evaluated by the tool of Hopkins Verbal Learning Test (HVLT) (20).

Only three studies (14, 15, 20) reported on QOL and no significant differences were observed between the PCI and non-PCI group.

## Discussion

This meta-analysis pooled seven updated randomized trials with 1,462 patients to reassess the role of PCI in NSCLC. The results confirmed the efficacy of PCI in terms of BM incidence reduction (RR = 0.37, *P* < 0.001) without a corresponding OS benefit for NSCLC (HR = 1.01, *P* = 0.74). The results of subgroup analysis suggested a beneficial role for PCI in prevention of BM regardless of pathology and local treatment. However, the positive role of PCI in reducing the risk of BM could only been observed in patients with good performance status.

To the best of our knowledge, this is the most recent meta-analysis with all available updated data from randomized controlled trials regarding the role of PCI for NSCLC. Previously, four meta-analyses (21–24) have been carried out to evaluate the efficacy of PCI for NSCLC with inconsistent results. Published reviews demonstrated the effect of PCI in the prevention of BM in NSCLC which are in line with our findings. However, with regard to effect of PCI on OS, previous reviews drew discordant conclusions. In the meta-analysis of Al Feghali and Lester (21, 22), PCI failed to prolong survival for NSCLC while Xie et al. (24) reported a detrimental effect on survival with PCI over observation. The meta-analysis by Xie et al. (24) included the RCT of Pottgen et al. (12), which was judged by our reviewers impossible to evaluate the effect of PCI on BM due to the study design that local treatment between the two arms was different. Furthermore, the meta-analysis by Xie et al. (24) didn't include the recent RCT of Li et al. (15) and De Ruysscher et al. (14) with proper study design and advanced brain imaging, staging and treatment modality. Recently, the trial RTOG 0214 with the largest sample size published its long follow-up result indicating that PCI could improve 5- and 10-years DFS but had no beneficial impact on OS (17). Including the updated data of RTOG 0214, our study revealed no beneficial effect of PCI on OS for NSCLC. The lack of OS benefit may be associated with absence of extra-cranial tumor control that result in death. Furthermore, brain MRI surveillance and early salvage brain radiotherapy (WBRT/SRT) may not compromise OS for patients randomized to observation group.

We further performed subgroup analysis to evaluate if PCI could reduce the BM risk for the subset with the different risks of BM. It has been recognized that patients with advanced tumor stage, non-squamous cell carcinoma histology, and younger age have a high propensity for CNS dissemination (25–27). Using available data from published trials, we performed subgroup analysis only by pathology, local treatment modality and performance status. Local treatment with prior surgery may be an indicator of relatively lower tumor stage. In our study, the treatment effects of PCI were similar between the subgroups by pathology (squamous cell carcinoma vs. non-squamous cell carcinoma) and prior treatment (prior surgery vs. no prior surgery). Interestingly, according to our subset analysis by performance status, PCI failed to reduce BM for patients with WHO 2–3. Performance status is a widely recognized prognostic factor for NSCLC as well as a predictor for BM in some studies. In the study of Sørensen et al., patients in the best performance group (Karnosfsky performance statue >60) had high risk for the development of BM (28). The relatively short survival in patients with poor performance status may contribute to decreased incidence of BM and therefore result in no significant benefit derived from PCI.

In addition to the efficacy of PCI, toxicities, and QOL are another major concern. Acute toxicities commonly include alopecia, headache, fatigue, nausea, and vomiting, which are generally manageable. The incidence of late non-neurologic sequelae, such as weakness and fatigue is relatively low (<5%). PCI can result in some neurocognitive function deterioration but has no effect on QOL. To preserve cognition for patients with PCI, there are several strategies. Firstly, the delivered PCI dose for NSCLC could be optimized. In limited stage small cell lung cancer, a lower dose of 25 Gy was non-inferior and correlated with less neurocognitive toxicity compared to a higher dose of 36 Gy (29). Secondly, reducing radiation dose to the hippocampus in the case of whole brain radiotherapy (WBRT) has been demonstrated to preserve cognitive function for patients with BM (30). Thirdly, use of neuroprotective drug Memantine concurrently with WBRT could delay time to cognitive function decline as well as decrease the rate of memory decline, executive function and processing speed (31).

Our meta-analysis has several strengths. The methodology of this meta-analysis is in line with the Cochrane Handbook of Systematic Reviews. Furthermore, this meta-analysis included the most recent data of included randomized trials. Besides, it is the only meta-analysis with subgroup analysis focusing on the impact of PCI on BM and identified patients with poor performance status not suitable for PCI.

Several limitations of the meta-analysis should be considered in interpreting our findings. Firstly, the studies included were heterogeneous with different study design and various patient populations and the analysis is not based on individual patient data. Secondly, we could not clarify the impact of PCI on OS of different subsets because the survival data was not available in most of the included trials. Finally, we did not rule out the risk of bias in individual studies, as the number of included articles was <10.

## Conclusions

Overall, our meta-analysis reveals that PCI significantly decreases BM incidence without a corresponding OS benefit. However, PCI fails to prevent BM for patients with poor performance status. PCI may impair neurocognitive function but have no impact on QOL. An individual patient data meta-analysis may identify patients with high propensity for BM that could achieve OS prolongation with PCI.

## Data Availability Statement

The datasets generated for this study are available on request to the corresponding author.

## Author Contributions

LL contributed to the study design, data collection, and analysis as well as writing of manuscript. TZ, QZ, and JC contributed to the data collection and data interpretation. XX and GL contributed to the revision of manuscript. All authors reviewed and approved the final manuscript.

### Conflict of Interest

The authors declare that the research was conducted in the absence of any commercial or financial relationships that could be construed as a potential conflict of interest.
