# Supplementary material for: The Role of Prophylactic Cranial Irradiation in Patients With Non-small Cell Lung Cancer: An Updated Systematic Review and Meta-Analysis
Source: Front Oncol. 2020 Jan 23;10:11. doi: 10.3389/fonc.2020.00011 (PMC6989473; doi:10.3389/fonc.2020.00011)
Supplement: Supplementary file 1 [file Table_1.DOCX]

**Supplementary**

**Search Strategy**

| **PUBMED**  (((((((NSCLC*[Title/Abstract]) OR Carcinoma, Non-Small-Cell Lung[MeSH Terms])) OR ((((lung*[Title/Abstract]) OR (bronch*[Title/Abstract]))) AND ((carcino*[Title/Abstract]) OR (cancer*[Title/Abstract]) OR (tumor*[Title/Abstract]) OR (tumour*[Title/Abstract]) OR (malignan*[Title/Abstract]) OR (oncol*[Title/Abstract]))))) AND ((((((((brain*[Title/Abstract]) OR (cran*[Title/Abstract]))) AND ((carcino*[Title/Abstract]) OR (cancer*[Title/Abstract]) OR (tumor*[Title/Abstract]) OR (tumour*[Title/Abstract]) OR (malignan*[Title/Abstract]) OR (oncol*[Title/Abstract]) OR (metasta*[[Title/Abstract]))) AND ((Radiotherapy[MeSH Terms]) OR (Radioth*[Title/Abstract]) OR (Radiat*[Title/Abstract])))) OR Cranial Irradiation[MeSH Terms]) OR Brain Neoplasms/radiotherapy[MeSH Terms])) AND (((((((Survival Analysis[MeSH Terms]) OR Radiation Effects[MeSH Terms]) OR Brain/Radiation Effects[MeSH Terms]) OR Survival[MeSH Terms]) OR Mortality[MeSH Terms]) OR Survival Rate[MeSH Terms]) OR Survival*[Title/Abstract])) AND ((RCT*[Title/Abstract]) OR random*[Title/Abstract]) |
| --- |
| **EMBASE**  ('carcinoma, non-small-cell lung'/exp OR nsclc*:ab,ti OR ((lung*:ab,ti OR bronch*:ab,ti) AND (carcino*:ab,ti OR cancer*:ab,ti OR tumor*:ab,ti OR tumour*:ab,ti OR malignan*:ab,ti OR oncol*:ab,ti))) AND ('skull irradiation'/exp OR 'brain neoplasms/radiotherapy' OR ((brain*:ab,ti OR cran*:ab,ti) AND (carcino*:ab,ti OR cancer*:ab,ti OR tumor*:ab,ti OR tumour*:ab,ti OR malignan*:ab,ti OR oncol*:ab,ti OR metasta*:ab,ti) AND ('radiotherapy'/exp OR radioth*:ab,ti OR radiat*:ab,ti))) AND ('survival analysis'/exp OR 'radiation effects'/exp OR 'survival'/exp OR 'mortality'/exp OR 'survival rate'/exp OR survival*:ab,ti) AND (rct*:ab,ti OR random*:ab,ti) |
| **Cochrane**  1 MeSh descriptor: [Carcinoma, Non-Small-Cell Lung] explode all trees  2 NSCLC*:ti,ab,kw  3 (lung*:ti,ab,kw) OR (bronch*:ti,ab,kw)  4 (carcino*:ti,ab,kw) OR (cancer*:ti,ab,kw) OR (tumor*:ti,ab,kw) OR (tumour*:ti,ab,kw) OR (malignan*:ti,ab,kw) OR (oncol*:ti,ab,kw)  5 #1 OR #2 OR (#3 AND #4)  6 Mesh descriptor:[Cranial Irradiation] explode all trees  7 (brain*:ti,ab,kw) OR (cran*:ti,ab,kw)  8 (carcino*:ti,ab,kw) OR (cancer*:ti,ab,kw) OR (tumor*:ti,ab,kw) OR (tumour*:ti,ab,kw) OR (malignan*:ti,ab,kw) OR (oncol*:ti,ab,kw) OR (metasta*:ti,ab,kw)  9 MeSh descriptor: [Radiotherapy] explode all trees  10 (Radioth*:ti,ab,kw) OR (Radiat*:ti,ab,kw)  11 #9 OR #10  12 #6 OR (#7 AND #8 AND #11)  13 MeSh descriptor: [Survival analysis] explode all trees  14 MeSh descriptor: [Radiation effects] explode all trees  15 MeSh descriptor: [Survival] explode all trees  16 MeSh descriptor: [Mortality] explode all trees  17 MeSh descriptor: [Survival rate] explode all trees  18 Survival*:ti,ab,kw  19 #13 OR #14 OR#15 OR#16 OR#17 OR#18  20 RCT*:ti,ab,kw  21 random*:ti,ab,kw  22 #20 OR #21  23 #5 AND #12 AND #19 AND #22 |
